# Efficient and Specific Detection of *Salmonella* in Food Samples Using a *stn*-Based Loop-Mediated Isothermal Amplification Method

**DOI:** 10.1155/2015/356401

**Published:** 2015-10-12

**Authors:** Mevaree Srisawat, Watanalai Panbangred

**Affiliations:** ^1^Department of Biotechnology, Faculty of Science, Mahidol University, Rama VI Road, Bangkok 10400, Thailand; ^2^Mahidol University-Osaka University Collaborative Research Center for Bioscience and Biotechnology (MU-OU:CRC), Faculty of Science, Mahidol University, Rama VI Road, Bangkok 10400, Thailand

## Abstract

The *Salmonella* enterotoxin (*stn*) gene exhibits high homology among *S. enterica* serovars and *S. bongori*. A set of 6 specific primers targeting the *stn* gene were designed for detection of *Salmonella* spp. using the loop-mediated isothermal amplification (LAMP) method. The primers amplified target sequences in all 102 strains of 87 serovars of *Salmonella* tested and no products were detected in 57 non-*Salmonella* strains. The detection limit in pure cultures was 5 fg DNA/reaction when amplified at 65°C for 25 min. The LAMP assay could detect *Salmonella* in artificially contaminated food samples as low as 220 cells/g of food without a preenrichment step. However, the sensitivity was increased 100-fold (~2 cells/g) following 5 hr preenrichment at 35°C. The LAMP technique, with a preenrichment step for 5 and 16 hr, was shown to give 100% specificity with food samples compared to the reference culture method in which 67 out of 90 food samples gave positive results. Different food matrixes did not interfere with LAMP detection which employed a simple boiling method for DNA template preparation. The results indicate that the LAMP method, targeting the *stn* gene, has great potential for detection of *Salmonella* in food samples with both high specificity and high sensitivity.

## 1. Introduction

*Salmonella* remains a leading cause of food poisoning in humans and is also a major foodborne pathogen worldwide [1, 2]. The genus* Salmonella* is a member of the Enterobacteriaceae family and is divided into two species,* S. enterica* and* S. bongori*. More than 2500 serovars of* Salmonella*, mostly in the species of* enterica*, have been reported [3, 4].* Salmonella* is usually transmitted to humans through consumption of contaminated food. Most often contaminated food is of animal origin (such as eggs, beef, poultry, and milk) but can also include water and vegetables [5, 6]. Due to the health risk and economic impacts of foodborne illness associated with* Salmonella*, more rapid methods with high sensitivity and specificity for* Salmonella* detection are still required.

The conventional microbiological method for the detection and identification of* Salmonella* in food samples requires multiple subculture steps, followed by biochemical and serological confirmation tests. This method is time consuming and labor intensive and typically requires 5 to 7 days depending on the biochemical test and serological confirmation utilized [7, 8]. Various molecular-based methods have been used to detect* Salmonella* and other pathogens due to their sensitivity and ability for rapid detection. Among these methods, PCR has been successfully established as a valuable method which offers the rapid, sensitive, and specific detection of the selected genes in various pathogens such as* Escherichia coli* O157:H7,* Listeria monocytogenes*,* Salmonella* spp., and* Shigella* spp. [7, 8]. Detection of a number of foodborne pathogens, such as* L. monocytogenes*,* Salmonella* spp., and* Shigella* spp., has utilized real-time PCR allowing for the rapid analysis of samples [9–11]. Despite these advantages, PCR methods require complicated procedures and expensive equipment, such as thermocyclers and electrophoresis units, which are not suitable for use in field conditions.

Loop-mediated isothermal amplification (LAMP) is a recently developed technique that shows promise for use under conditions in which standard laboratory equipment is not available. Advantages of the LAMP technique include ease of operation, a high degree of specificity, and rapid and simple procedures compared to PCR methods. Detection of LAMP products is also suitable to field conditions as gel electrophoresis is not required [12]. The LAMP method produces large amounts of pyrophosphate, a by-product of DNA amplification, which can easily be detected by monitoring turbidity or fluorescence [13–15]. In addition, the presence of nontarget DNA and inhibitors in the LAMP reaction has been shown to not affect the amplification results [13]. This powerful technique with reduced rates of false positives and inhibition should be a viable tool for the detection of specific pathogens in food samples, since high amounts of nontarget DNA from many food ingredients as well as several inhibitors are usually present.

The aim of this study is to develop a LAMP assay that can be applied to the* Salmonella* enterotoxin (*stn*) gene for the detection of* Salmonella* in food samples. The simple method of DNA preparation from food samples and the sensitivity and the specificity of LAMP detection procedure were also described.

## 2. Materials and Methods

### 2.1. Bacterial Strains and Culture Conditions

One hundred and fifty-nine strains, including 102 strains (87 serovars) of* Salmonella enterica* and 57 non-*Salmonella* strains in the family Enterobacteriaceae, were obtained from World Health Organization National* Salmonella* and* Shigella* Center, National Institute of Health, Department of Medical Sciences, Ministry of Public Health, Nonthaburi, Thailand. All strains are listed in Supplementary Table S1 in Supplementary material, available online at http://dx.doi.org/10.1155/2015/356401.* Salmonella enterica* subsp.* enterica* serovar Typhimurium ATCC 23566 was used as the reference strain. All of bacterial cultures were stored in 15% (v/v) glycerol at −80°C. For cultivation,* Salmonella* spp. were subcultured on xylose lysine deoxycholate agar medium (XLD; Merck). Non-*Salmonella* bacterial strains were subcultured on Luria-Bertani agar (LA) plate (10 g tryptone; 5 g yeast extract; 10 g NaCl; 15 g agar; and H_2_O to 1000 mL) and incubated at 37°C overnight.

### 2.2. Sequence Comparison of the* stn* Gene

The* stn* gene sequences from* Salmonella* and non-*Salmonella* species of the Enterobacteriaceae family were obtained from the GenBank database. Pairwise sequence comparisons were performed using the EMBOSS Needle tool program (EMBL-EBI). Sequences were aligned using the MUSCLE multisequence alignment program. Phylogenetic relationships and evolutionary history were inferred from this alignment using the MEGA5 software program and the Neighbor-Joining method, respectively. The bootstrap consensus tree from 2000 replicates was taken to represent the evolutionary history of the taxa analyzed.

### 2.3. Primer Design for LAMP

A set of four primers comprising two inner primers (FIP and BIP) and two outer primers (F3 and B3) that recognize six distinct sequences within the* Salmonella* enterotoxin (*stn*) gene of* Salmonella* Typhimurium (GenBank Accession Number L16014) were designed. To compare amplification efficiency, loop primers, LF and LB (between the F1/F2 region and B1/B2 region, resp.), were also designed to check their role in increasing the sensitivity of detection. A set of LAMP primers was designed using the PrimerExplorer V4, LAMP primer design program (http://primerexplorer.jp/e/index.html). The designed primers were compared with the NCBI sequence database to confirm the specificity of the primers. The nucleotide sequences and annealing portions of each primer are shown in Table 1 and Supplementary Figure S1, respectively.

### 2.4. LAMP Reaction and LAMP Product Detection

The LAMP reaction was performed in a total volume of 25 *μ*L containing the following components (final concentration): 1.6 *μ*M each of FIP and BIP primers, 0.2 *μ*M each of F3 and B3 primers, 0.8 *μ*M each of LF and LB primers (in the same LAMP reaction), 1.6 mM of deoxyribonucleotide triphosphate mixture (dNTPs), 1 M betaine (Sigma, B2629, St. Louis, USA), 6 mM MgSO_4_, 1x thermopol buffer (New England Biolabs, B9004S, Beverly, USA), 1 *μ*L (8 U) of* B*st DNA polymerase large fragment (New England Biolabs, M0275S, Beverly, USA), and 5 *μ*L of DNA template solution. The reaction temperature was optimized by incubating the reaction mixture under isothermal conditions between 60 and 70°C for 60 min. The reaction time was optimized by varying the time in each reaction from 10 to 60 min, at 5 min intervals in each condition at the optimal temperature (65°C). The reaction was terminated by heating at 80°C for 5 min.* S*. Typhimurium ATCC 23566 cells or its isolated DNA (1 ng/reaction) was used as positive controls. LF and LB primers were added to determine if these primers increased amplification efficacy. For analysis of the LAMP products, the turbidity resulting from the white precipitate of magnesium pyrophosphate in the mixture was observed by eye and confirmed by monitoring the formation of a green color under normal light following addition of SYBR green. The negative reaction was orange in color. LAMP products (2.5 *μ*L) were also analyzed by electrophoresis using 2% agarose gel, stained with ethidium bromide, and visualized using a UV transilluminator.

### 2.5. DNA Template Preparation and Sensitivity of LAMP Detection

DNA template for all bacterial strains was prepared using the boiling method [16].* Salmonella* DNA templates were prepared from 1.5 mL of overnight cultures grown in lactose broth incubated at 37°C. Cells were harvested and washed twice and then resuspended in 100 *μ*L of TE buffer. Lysis was performed by boiling at 100°C for 10 min, and supernatant was collected. Non-*Salmonella* strains were grown in LB broth and DNA templates were prepared as described above. To determine the minimum DNA concentration required for the LAMP reaction template DNA from* S*. Typhimurium ATCC 23566 was prepared following the total DNA isolation procedure as described by Wilson [17]. Genomic DNA at 15.625 pg, 3.125 pg, 625 fg, 125 fg, 25 fg, 5 fg, and 1 fg DNA/reaction was used as DNA templates in a total volume of 25 *μ*L. To determine the minimal cell number,* S*. Typhimurium ATCC 23566 was cultured in lactose broth and incubated at 37°C overnight (16 hr), then subcultured (1% inoculum) in freshly prepared lactose broth, and further incubated at 35°C for 5 hr with shaking. LAMP assays were performed using cell numbers ranging from 0 to 1000 cells/reaction and DNA template was prepared by the boiling method as described above. The numbers of cells were monitored by the plate count technique on XLD agar plate after incubation at 37°C for 48 hr.

### 2.6. *Salmonella* Detection in Artificially Contaminated Food

A single colony of* S*. Typhimurium ATCC 23566 was picked from XLD agar plate and was inoculated into lactose broth and incubated at 37°C overnight (16 hr). The number of viable cells was obtained using the plate count technique on XLD agar plate with incubation at 37°C for 48 hr. The plate count was performed in triplicate. Minced pork meat was decontaminated by autoclaving at 121°C for use as a sterile food sample [18]. The autoclaved food sample (25 g in 225 mL of lactose broth) was artificially contaminated by spiking with 1000 *μ*L of appropriate dilutions of* S*. Typhimurium ATCC 23566 to achieve 5.5 × 10^6^–55 cells/25 g of food sample. The inoculated food sample was then mixed with 225 mL lactose broth and enriched at 35°C for 5 hr. 1.5 mL of inoculated food sample was taken out at 0 and 5 hr time points and stood without shaking for 5 min to allow particulate matter to settle. One mL of the upper portion was collected and centrifuged for 5 min at 10000 rpm. The pellet was washed twice with 500 *μ*L of TE buffer and resuspended in 100 *μ*L of TE buffer. DNA was extracted using the simple boiling method as described above. The supernatant (5 *μ*L) was also directly used as the DNA template for LAMP amplification. These experiments were performed in triplicate. Autoclaved minced pork meat without* Salmonella* inoculation was included in every experiment as a negative control.

### 2.7. Detection of* Salmonella* in Naturally Contaminated Food

To determine the validity and reliability of LAMP detection of the* stn* gene for* Salmonella* identification in food samples a comparative study between LAMP and the reference culture method was performed. Various kinds of foods with possible naturally occurring* Salmonella* contamination were investigated. A total of 90 food samples, 30 each of minced pork meat, chicken meat, and fresh vegetables, were purchased randomly in a local market in Bangkok. All food samples were transported to the laboratory in an ice box and were examined immediately after purchase. Each food sample (25 g) was homogenized in 225 mL of lactose broth. The mixture was incubated at 35°C and 1.5 mL was collected for DNA extraction after 5 and 16 hr of incubation. DNA was then prepared from food samples for use in LAMP detection. The reference culture method was performed in parallel as described in the* Bacteriological Analytical Manual* (*BAM 8th Edition*) [19]. The performance indicators for qualitative methods and efficacy of LAMP method in comparison with the reference culture method for* Salmonella* detection in food samples were calculated using the method of Galen [20].

## 3. Results

### 3.1. *stn* Gene Sequence Data Analysis

A total of 77 putative* stn* whole gene sequences from Enterobacteriaceae strains were collected from GenBank. Similarities of* stn* among various genera are listed in Table 2. The nucleotide sequence of* stn* among 16* Salmonella enterica *strains shows 96.9–100% homology. Only one* stn* gene sequence for* S. bongori* was available which exhibited 83.8–84.7% homology to the 16* stn* sequences from* S. enterica* (Table 2). The* stn* sequence from* Salmonella* exhibited 57.0% to 74.0% sequence homology to* stn* genes from other genera, with the highest homology to that of* Citrobacter* at 73.2% to 74.0%. The phylogenetic relationships of* stn* from 12 serovars, 17 strains (16 for* S. enterica* and one for* S. bongori*) in the genus* Salmonella* with other genera in the family Enterobacteriaceae were determined. The dendrograms inferred from the* stn* sequence alignment using the Neighbor-Joining method (Supplementary Figure S2) and Maximum Likelihood method yielded similar topologies in which* Salmonella* formed individual clusters. The toxin gene of* S. aureus* was used as the out group. The data suggested that the* stn* gene should provide a genus-specific target sequence for detection of* Salmonella.*

### 3.2. Optimal Condition for the LAMP

The optimal temperature for LAMP assay was determined by varying the temperature between 60 and 70°C for 60 min in 1°C increments using purified DNA template. The LAMP products amplified between 64 and 66°C showed clear and distinct DNA bands with higher density compared to other samples. Therefore, 65°C was set as an optimal temperature. Reaction times, 5–60 min at 5 min intervals, were analyzed with the reaction temperature at 65°C using 0.8 *μ*M each of LF and LB loop primers. LAMP products were detected as early at 15 min (Supplementary Figure S3(B), lane 3) but the clear and intense bands were observed at 20 min (Supplementary Figure S3(B), lane 4). In the absence of loop primers clear and dense bands were observed after 35 min (Supplementary Figure S3(A), lane 7). Therefore, the optimal condition for the* stn* LAMP assay was set at 65°C for 25 min with 0.8 *μ*M each of loop primers, 1.6 *μ*M each of FIP and BIP primers, and 0.2 *μ*M each of outer primers. The effect of various LAMP reagent concentrations (MgCl_2_, dNTPs, betaine, and* Bst* DNA polymerase) was also studied (data not shown). The optimal conditions for the highest sensitivity for* Salmonella stn* gene detection using LAMP are summarized in Table 3.

### 3.3. Specificity of LAMP

The results for the specificity test of the* stn* LAMP assay are shown in Supplementary Table S1. In this analysis all of 102 strains of 87 serovars of* Salmonella* gave positive results. In contrast, the 57 non-*Salmonella* strains in family Enterobacteriaceae including 6 strains of* Citrobacter* examined by LAMP were negative. Product formation in the LAMP assays was monitored by observing the presence of white turbidity and by observing green color following addition of SYBR green in the reaction mixture. The presence of amplified products was also confirmed using gel electrophoresis with 2% agarose gels. No amplified products were observed in LAMP reactions lacking* Salmonella* DNA demonstrating that amplification of the* stn* gene was highly specific for* Salmonella* detection. Examples of LAMP assays using DNA template from some* Salmonella* serovars and other enteric bacteria are shown in Supplementary Figure S4.

### 3.4. Sensitivity of Detection

Analysis using purified DNA prepared from* S*. Typhimurium ATCC 23566 revealed that the lowest DNA concentration that could promote* stn* amplification by LAMP (at 65°C, 25 min with loop primers) was 5 fg DNA/25 *μ*L reaction (Supplementary Figure S5(A)). Similar analysis using DNA prepared from whole cells by boiling method indicates that a minimum of 1 cell/reaction was sufficient to give a positive result with the* stn* LAMP assay under these conditions (Supplementary Figure S5(B)).

### 3.5. Detection of* Salmonella* in Artificially and Naturally Contaminated Food Samples

All six samples of autoclaved minced pork meat (25 g) in 225 mL of lactose broth inoculated with 5.5 × 10^6^–55 cells of* S*. Typhimurium ATCC 23566 gave positive result. In contrast, uninoculated meat samples were negative. The detection limit of* Salmonella* in artificially contaminated food using the LAMP assay was 5.5 × 10^3^ cells/250 mL (220 cells/g of food sample) without an enrichment step (at 0 hr), as shown in Table 4. The sensitivity increased to 55 cells/250 mL (2 cells/g of food sample) after incubation of the inoculated food sample for 5 hr at 35°C prior to LAMP analysis (Table 4). A total of 90 food samples were analyzed using both the reference culture method according to BAM and the LAMP method. This analysis used 30 samples each of minced pork meat, chicken meat, and fresh vegetables enriched for either 5 or 16 hr. The results (data not shown) indicated that 67 samples were positive and 23 samples were negative by both methods after enrichment for 5 hr. The meat samples gave higher positive results (28 and 25 for minced pork meat and chicken meat, resp.) than those of fresh vegetable (14 out of 30 samples) (data not shown). Preenrichment times of 5 hr and 16 hr gave similar results (data not shown). The overall relative diagnostic specificity and accuracy were judged to be 100% as no false-negative or false-positive results were obtained from the LAMP assay compared to the culture method.

## 4. Discussion

The LAMP method has been utilized to efficiently detect several foodborne pathogens such as* Staphylococcus aureus* [21],* Bacillus cereus* [22],* Vibrio vulnificus* [23], and* Salmonella*. Previous reports for LAMP detection of* Salmonella* utilized several gene targets including* invA* [14, 16, 24, 25],* phoP* [15],* Sdf I* [12], and* IS200/IS1351* [26]. More recently, a* fimY*-based LAMP assay has been used to detect 80* Salmonella* strains of 24 serovars [27]. In our study, we targeted the* stn* (*Salmonella* enterotoxin gene) which is widely distributed among* Salmonella* serovars and has been identified in all 95 strains of* Salmonella* [28]. The* stn* gene exhibits high nucleotide sequence homology among* Salmonella* strains but low homology to the corresponding gene among other closely related enteric bacteria. It has also been reported that* stn* gene amplification can be effectively used to specifically detect 52 strains of* S. enterica* and 2 strains of* S. bongori* without cross-reacting with several common intestinal strains [29]. Based on our results and previous findings it can be established that* stn* is a suitable target gene for direct detection of* Salmonella* in biological samples.

The* stn* gene from* S. enterica* encodes a protein of approximately 29 kDa, utilizing a TTG start codon, and contains a portion of the conserved motif found in other protein toxins with ADP ribosylation activity [30]. The results of LAMP assay using the* stn* gene in our study confirmed that it was an appropriate target to specifically detect* Salmonella* strains without cross-reaction with other closely related enteric bacteria from a complex matrix, such as food samples. We did not include* S. bongori* in our analysis; however,* S. bongori* displays 88% sequence identity with* S. enterica stn*. Primers targeting the* stn* gene efficiently amplify both* S. enterica* and* S. bongori* in real-time PCR [31] and PCR [29] analysis, suggesting that the LAMP procedure should also be applicable for detection of* S. bongori*. The available* stn* gene sequences of* Enterobacter* and* Citrobacter* strains in GenBank database, respectively, showed 66.9–67.8% and 73.2–74.0% similarity with* stn* gene sequence from 16* Salmonella* strains. However, when 5 strains of* Enterobacter* and 6 strains of* Citrobacter* were used in the* stn* LAMP test with* Salmonella* specific primers, no positive results were detected. The lack of false-positive results with non-*Salmonella* strains suggests that* stn* can be utilized as specific target gene for* Salmonella* detection.

The presence of loop primers in reaction mixtures reduced the time required for amplification of* stn* gene to 25 min and provided high sensitivity, allowing the detection of only one* Salmonella* cell in artificially contaminated food samples. The LAMP assay targeting* invA* gene (also with loop primers) can detect pure culture of* Salmonella* at 2.8 cells/reaction tube [14]. In other studies, the LAMP method targeting* phoP* gene for detecting* Salmonella* in 20 hr preenrichment food samples shows the lowest limit of detection at 35 cells/reaction tube. Hence, our optimized LAMP method targeting* stn* gene provided more sensitive detection limit compared to* invA* and* phoP* genes. The gene sequence similarities of* phoP* [15] between different genera of enteric bacteria compared to* Salmonella* are quite high at 70.8–85.9%, whereas, for* stn*, sequence similarities among these bacteria were only 57.6–73.9% (Table 4). In both studies on* phoP* and* stn*,* Citrobacter* is the genus most similar to* Salmonella*, showing 85.6–85.9% and 73.9% homology for* phoP* and* stn* genes, respectively. The low sequence similarity of* stn* genes among other enteric bacteria compared to* Salmonella* may provide more specific detection of* Salmonella* in different kinds of food samples in which various enteric bacteria are simultaneously present. Therefore, these assay conditions targeting the* stn* gene constitute a valuable procedure for the rapid detection of* Salmonella* in food samples.

## 5. Conclusion

*Salmonella* enterotoxin (*stn*) gene is highly conserved among* S. enterica* serovars and* S. bongori* [28, 29, 31–33]. The six primers designed from* stn* gene could specifically detect 87 serovars of* S. enterica* (102 strains) without cross-reacting with 57 non-*Salmonella* strains, including* C. diversus* (3 strains) and* C. freundii* (3 strains).

The* stn* amplification using the LAMP procedure was shown to be highly accurate for detection of* Salmonella* in various food matrices without cross-reacting with other contaminated bacteria in the food samples, even from other closely related enteric bacteria. This LAMP assay using* stn* as a target gene has the potential as a rapid method for detection of* Salmonella* with high sensitivity and specificity and could be used as a method of choice in diagnostic food laboratories.

## Supplementary Material

The supplementary materials include 1 Table and 5 Figures. Table S1 describes the name list of specificity test for stn detection by LAMP of 102 and 57 strains of Salmonella and non-Salmonella bacteria. The 5 Supplementary Figures show the nucleotide sequence of stn and primers annealing sites (Figure S1), Phylogenetic tree of stn sequences of various bacterial strains of the family Enterobacteriaceae (Figure S2). In Figures S3, S4 and S5 show electrophoresis patterns of LAMP amplified products with and without loop primers, specificity for stn among various strains of Enterobacteriaceae and sensitivity of stn LAMP assays using purified DNA or DNA from boiled cells, respectively.

## Figures and Tables

**Table 1:** Oligonucleotide primer sequences used in *stn* LAMP analysis.

Primer	Primer type	Sequence (5′-3′)
F3	Forward outer (F3)	5′ ACCAGATTCAGGGAGTGAGT 3′
B3	Backward outer (B3)	5′ CGCGCACGAAATTCGTAAC 3′
FIP	Forward-inner (F1c-F2)	5′ ACCGGGTGGTAAGCGAATTGC-
		GAGGTTAACCGTCTGGAGC 3′
BIP	Backward-inner (B1c-B2)	5′ TCGGCCTCTTTGGCCATCAC-
		TGGCGAAATACTTTGCCGAG 3′
Loop F	Loop forward (LF)	5′ TGGTAAAGCCCGCGCATCTG 3′
Loop B	Loop backward (LB)	5′ GCGCCAGTTCATGCGACTCG 3′

Note: primers for LAMP were designed using the *stn* gene (GenBank Accession number L16014) from *Salmonella enterica* subsp. *enterica* serovar Typhimurium.

**Table 2:** The *stn* gene sequence similarity values among 77 bacterial strains of Enterobacteriaceae.

Genera	Similarity to					
	*Klebsiella *	*Escherichia *	*Shigella *	*Enterobacter *	*Citrobacter *	*Salmonella enterica *
*Klebsiella* (2)	71.6–100					
*Escherichia* (49)^a^	68.3–73.7	89–100				
*Shigella* (8)	67.1–73.0	91.5–98.0	94.9–100			
*Enterobacter* (1)	64.8–67.0	68.1–68.8	67.5–69.2	100		
*Citrobacter* (1)	60.0–63.9	63.4–64.2	63.5–65.2	70.1	100	
*Salmonella enterica* (16)	57.0–60.4	61.6–66.7	63.0–64.5	66.9–67.8	73.2–74.0	96.9–100
*Salmonella bongori* (1)	57.6–60.4	60.0–66.2	62.6–64.5	67.8	73.9	83.8–84.7

^a^The number in parenthesis is the number of sequences collected from the NCBI database.

**Table 3:** Optimization of LAMP assay for *Salmonella* detection by targeting the *stn *gene.

Conditions	The tested range	Difference	Good yield	Selected condition^a^
Temperature	60–65°C	1°C	64–66°C	65°C
Time	0–60 min	5 min	15–60 min	25 min
Loop primers	0.5–1.0 *μ*M	0.1 *μ*M	0.8–1.0 *μ*M	0.8 *μ*M
Outer primers	0.1–0.5 *μ*M	0.1 *μ*M	0.2–0.5 *μ*M	0.2 *μ*M
MgSO_4_	4–8 mM	0.5 mM	6–7.5 mM	7 mM
dNTPs	0.8–2.4 mM	0.2 mM	1.2–1.8 mM	1.6 mM
Betaine	0.5–1.5 M	0.1 M	0.6–1.5 M	1.0 M
*Bst* DNA polymerase	4–12 units	1 unit	8–12 units	8 units

^a^The final concentrations of reagents or temperature and times used in LAMP analysis.

The concentration of FIP and BIP primers are fixed at 1.6 *μ*M.

**Table 4:** Sensitivity for LAMP detection of *Salmonella* in artificially contaminated food samples using DNA templates prepared by the boiling method.

Total cells per^a^				Enrichment time	
250 mL of food suspension	Gram of food sample	1 mL of food suspension^b^	LAMP reaction^c^	(0 hr)	(5 hr)
5.5 × 10^6^	2.2 × 10^5^	2.2 × 10^4^	1.1 × 10^3^	+	+
5.5 × 10^5^	2.2 × 10^4^	2.2 × 10^3^	1.1 × 10^2^	+	+
5.5 × 10^4^	2.2 × 10^3^	2.2 × 10^2^	1.1 × 10^1^	+	+
5.5 × 10^3^	2.2 × 10^2^	2.2 × 10	1.1	+	+
5.5 × 10^2^	2.2 × 10	2.2	0	−	+
5.5 × 10^1^	2.2	0.2	0	−	+

^a^The number of cells at 0 hr (before enrichment) using 25 g of food sample in 225 mL of lactose broth.

^b^One mL of food sample was used to prepare DNA template by the boiling method. Samples were resuspended in 100 *μ*L of TE buffer and used as DNA template.

^c^5 *μ*L of DNA template was used in a 25 *μ*L LAMP reaction. Loop primers were included in the reaction mixture.
