# Supplementary material for: Efficient and Specific Detection of Salmonella in Food Samples Using a stn-Based Loop-Mediated Isothermal Amplification Method
Source: Biomed Res Int. 2015 Oct 12;2015:356401. doi: 10.1155/2015/356401 (PMC4620276; doi:10.1155/2015/356401)
Supplement: Supplementary file 1 — The supplementary materials include 1 Table and 5 Figures. Table S1 describes the name list of specificity test for stn detection by LAMP of 102 and 57 strains of Salmonella and non-Salmonella bacteria. The 5 Supplementary Figures show the nucleotide sequence of stn and primers annealing sites (Figure S1), Phylogenetic tree of stn sequences of various bacterial strains of the family Enterobacteriaceae (Figure S2). In Figures S3, S4 and S5 show electrophoresis patterns of LAMP amplified products with and without loop primers, specificity for stn among various strains of Enterobacteriaceae and sensitivity of stn LAMP assays using purified DNA or DNA from boiled cells, respectively. [file 356401.f1.pdf]

Supplementary Material

The Supplementary Materials include 1 supplementary table and 5 supplementary figures as follows.

Supplementary Table S1: Specificity test for *stn* LAMP assays.

Supplementary Figure S1: The nucleotide sequence of *stn* gene and primers used for LAMP assay, arrows indicate the position and direction of DNA synthesis.

Supplementary Figure S2: Phylogenetic tree derived from the entire *stn* sequence of members of various genera of the family *Enterobateriaceae* determined by the Neighbor-Joining method. Numbers within the dendrogram indicate the occurrence (%) of branching in the 2000 bootstapped tree. Branches corresponding to partitions reproduced in less than 50% of bootstrap replicates are collapsed.

Supplementary Figure S3: Electrophoresis analysis of *stn* LAMP amplified products without loop primer (3A) and with loop primers (3B) using different reaction times. Lane M: 100 bp DNA ladder as size markers, lanes 1-12: LAMP carried out for 5, 10, 15, 20, 25, 30, 35, 40, 45, 50, 55 and 60 min, respectively; and lane 13: negative control (absence of DNA template).

Supplementary Figure S4: Specificity test for the *stn* LAMP assay. Lane M: 100 bp DNA ladder as size marker, lane 1: *S.* Anatum; lane 2: *S.* Amsterdam; lane 3: *S.* Bangkok; lane 4: *S.* Choleraesuis; lane 5: *S.* Derby; lane 6: *S.* Enteritidis, lane 7: *S.* Paratyphi, lane 8: *Citrobacter frundii,* lane 9: *Eschericia coli* K12, lane 10: *Enterococcus faecalis,* lane 11 *Enterobacter tarda*, lane 12: *Proteus vulgaris,* lane 13: *S.* Typhimurium ATCC 23566 as positive control, 14: negative control (no DNA template).

Supplementary Figure S5: Sensitivity of *stn* LAMP assays using purified DNA (A) and DNA from boiled cells (B). (A) Amount of purified DNA needed for detection of *stn* LAMP products. Lane M: 100 bp DNA ladder as size marker; lanes 1-6, reaction carried out using 5

fold-serial dilutions of standard genomic DNA at 3125 fg, 625 fg, 125 fg, 25 fg, 5 fg and 1 fg DNA/reaction, respectively; lane 7, negative control (no DNA template). (B) The number of *Salmonella* cells required for detection of *stn* by LAMP. Lane M: 100 bp DNA ladder as size marker; lanes 1-4, reaction carried out using DNA prepared directly from boil cells of 10^3, 10^2, 10 and 1 cells/reaction, respectively; lane 5, negative control (no DNA template).

SUPPLEMENTARY TABLE S1: Specificity test for *stn* LAMP assays.

Microorganisms		No. of strains	LAMP
Salmonella enterica subsp. *enterica* Serovar (I)	Aberdeen	1	+
	Abony	1	+
	Agona	1	+
	Ajana	1	+
	Alachua	1	+
	Altona	1	+
	Amsterdam	1	+
	Anatum	3	+
	Augustenborg	1	+
	Bangkok	1	+
	Bareilly	1	+
	Berta	1	+
	Blockley	1	+
	Bovismorbificans	1	+
	Braenderup	1	+
	Brunei	1	+
	Cerro	1	+
	Chester	1	+
	Choleraesuis	1	+
	Circhow	1	+
	Corvallis	1	+
	Cubana	1	+
	Derby	1	+
	Dublin	1	+
	Emek	1	+
	Enteritidis	2	+
	Falkensee	1	+
	Farmsen	1	+
	Gallinarum	1	+
	Gera	1	+
	Give	1	+
	Hadar	1	+
	Havana	1	+
	Heidelberg	1	+
	Hvittingfoss	1	+
	Infantis	1	+
	Isangi	1	+

SUPPLEMENTARY TABLE S1: Specificity test for *stn* LAMP assays (continued).

Microorganisms		No. of strains	LAMP
Salmonella enterica subsp. *enterica* Serovar (I)	Javiana	1	+
	Kedougou	1	+
	Kentucky	1	+
	Krefeld	3	+
	Langensalza	1	+
	Lexington	1	+
	Liverpool	1	+
	Livingstone	1	+
	London	1	+
	Manhattan	1	+
	Mbandaka	3	+
	Molade	1	+
	Montevideo	1	+
	Muenchen	1	+
	Newport	1	+
	Ohio	1	+
	Orion	2	+
	Oslo	1	+
	Panama	1	+
	Paratyphi A	1	+
	Paratyphi B	2	+
	Poona	1	+
	Potsdam	1	+
	Rissen	1	+
	Rubislaw	1	+
	Saintpaul	1	+
	Sandiego	1	+
	Schwarzengrund	1	+
	Senftenberg	1	+
	Singapore	1	+
	Stanley	1	+
	Tennessee	1	+
	Thompson	1	+
	Typhi	1	+
	Typhimurium	3	+
	Urbana	1	+
	Virchow	2	+
	Wandswarth	1	+

SUPPLEMENTARY TABLE S1: Specificity test for *stn* LAMP assays (continued).

Microorganisms	No. of strains	LAMP
Salmonella enterica subsp. *enterica* Serovar (I) Waycross	1	+
Welikade	1	+
Weltevreden	2	+
Westhampton	1	+
Wilmington	1	+
Worthington	1	+
Yerba	1	+
Zanzibar	1	+
Salmonella enterica subsp. *salamae* (II)	3	+
Salmonella enterica subsp. *arizonae* (IIIa)	1	+
Salmonella enterica subsp. *diarizonae* (IIIb)	1	+
Salmonella enterica subsp. *houtenae* (IV)	1	+
Citrobacter diversus	3	-
Citrobacter frundii	3	-
Enterobacter tarda	1	-
Enterobacter aerugenes	1	-
Enterobacter cloacae	3	-
Enterococcus faecalis	1	-
Escherichia coli	17	-
Klebsiella ozaenae	1	-
Klebsiella pneumoniae	10	-
Morganella morganii	1	-
Proteus mirabilis	1	-
Proteus rettegeri	1	-
Proteus vulgaris	1	-
Serratia macescens	1	-
Shigella boydii	1	-
Shigella flexneri	6	-
Shigella sonnei	4	-

Note: + = Positive amplification

- = Negative amplification

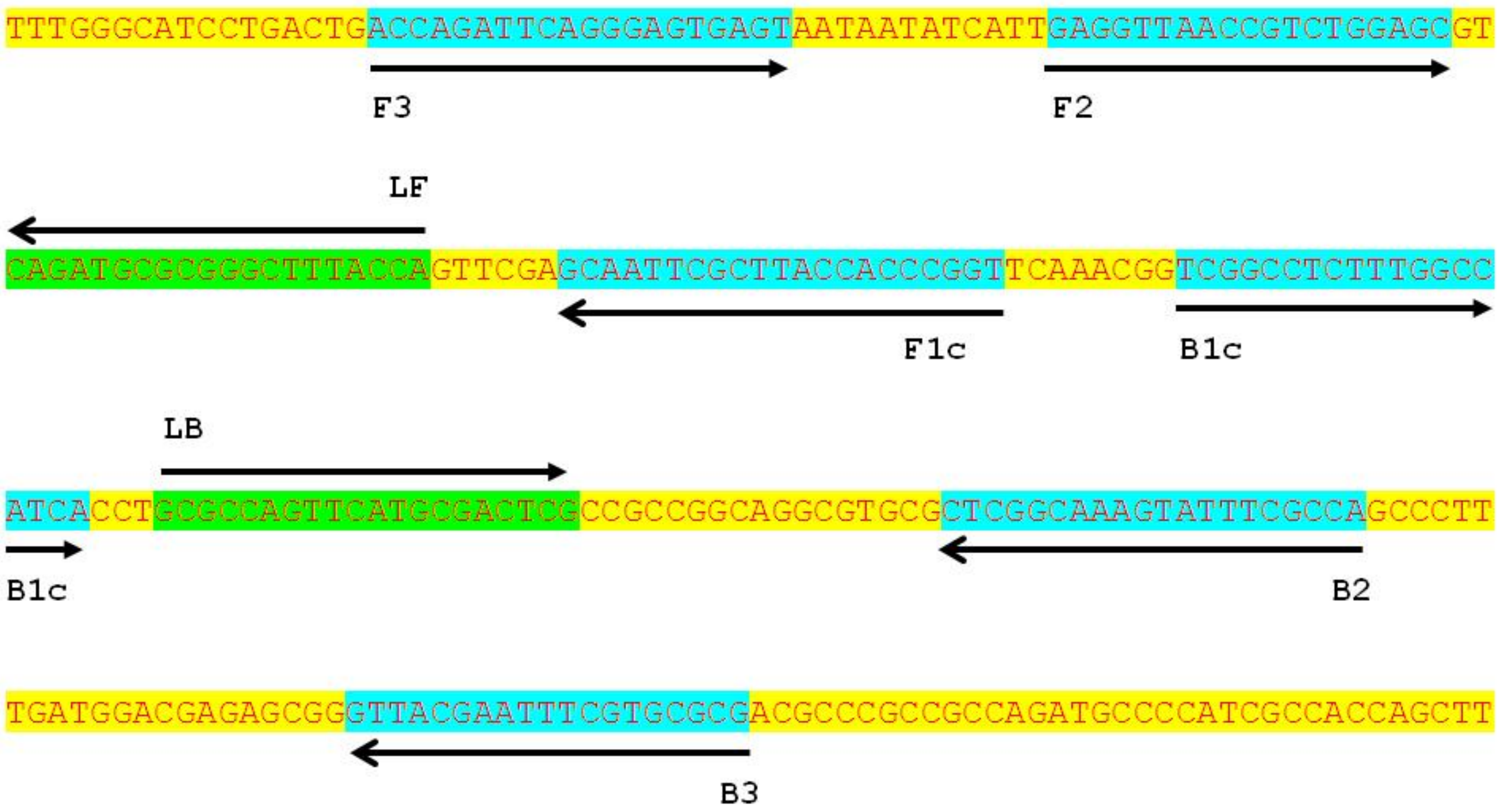
TTTGGGCATCCTGACTGACCAGATTCAGGGAGTGAGTAATAATATCATTGAGGTTAACCGTCTGGAGCGT
F3
F2
LF
CAGATGCGCGGGCTTTACCAGTTCGAGCAATTCGCTTACCACCCGGTTCAAACGGTCGGCCTCTTTGGCC
F1c
B1c
LB
ATCACCTGCGCCAGTTCATGCGACTCGCCGCCGGCAGGCGTGCGCTCGGCAAAGTATTTCGCCAGCCCTT
B1c
B2
TGATGGACGAGAGCGGGTTACGAATTTCGTGCGCGACGCCCGCCGCCAGATGCCCCATCGCCACCAGCTT
B3

FIGURE S1

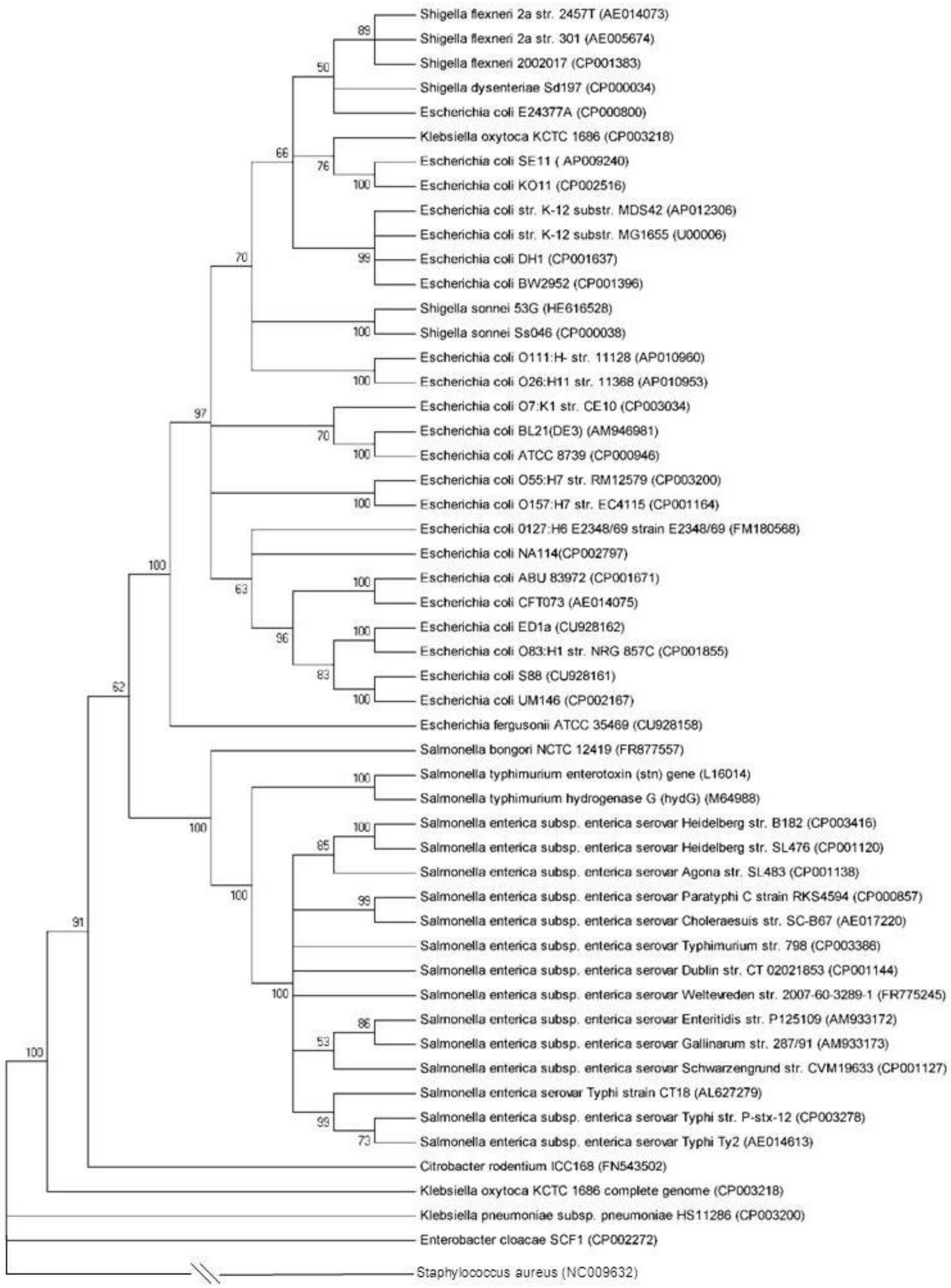
Shigella flexneri 2a str. 2457T (AE014073)
Shigella flexneri 2a str. 301 (AE005674)
Shigella flexneri 2002017 (CP001383)
Shigella dysenteriae Sd197 (CP000034)
Escherichia coli E24377A (CP000800)
Klebsiella oxytoca KCTC 1686 (CP003218)
Escherichia coli SE11 (AP009240)
Escherichia coli KO11 (CP002516)
Escherichia coli str. K-12 substr. MDS42 (AP012306)
Escherichia coli str. K-12 substr. MG1655 (U00006)
Escherichia coli DH1 (CP001637)
Escherichia coli BW2952 (CP001396)
Shigella sonnei 53G (HE616528)
Shigella sonnei Ss046 (CP000038)
Escherichia coli O111:H- str. 11128 (AP010960)
Escherichia coli O26:H11 str. 11368 (AP010953)
Escherichia coli O7:K1 str. CE10 (CP003034)
Escherichia coli BL21(DE3) (AM946981)
Escherichia coli ATCC 8739 (CP000946)
Escherichia coli O55:H7 str. RM12579 (CP003200)
Escherichia coli O157:H7 str. EC4115 (CP001164)
Escherichia coli 0127:H6 E2348/69 strain E2348/69 (FM180568)
Escherichia coli NA114(CP002797)
Escherichia coli ABU 83972 (CP001671)
Escherichia coli CFT073 (AE014075)
Escherichia coli ED1a (CU928162)
Escherichia coli O83:H1 str. NRG 857C (CP001855)
Escherichia coli S88 (CU928161)
Escherichia coli UM146 (CP002167)
Escherichia fergusonii ATCC 35469 (CU928158)
Salmonella bongori NCTC 12419 (FR877557)
Salmonella typhimurium enterotoxin (stn) gene (L16014)
Salmonella typhimurium hydrogenase G (hydG) (M64988)
Salmonella enterica subsp. enterica serovar Heidelberg str. B182 (CP003416)
Salmonella enterica subsp. enterica serovar Heidelberg str. SL476 (CP001120)
Salmonella enterica subsp. enterica serovar Agona str. SL483 (CP001138)
Salmonella enterica subsp. enterica serovar Paratyphi C strain RKS4594 (CP000857)
Salmonella enterica subsp. enterica serovar Choleraesuis str. SC-B67 (AE017220)
Salmonella enterica subsp. enterica serovar Typhimurium str. 798 (CP003386)
Salmonella enterica subsp. enterica serovar Dublin str. CT 02021853 (CP001144)
Salmonella enterica subsp. enterica serovar Weltevreden str. 2007-60-3289-1 (FR775245)
Salmonella enterica subsp. enterica serovar Enteritidis str. P125109 (AM933172)
Salmonella enterica subsp. enterica serovar Gallinarum str. 287/91 (AM933173)
Salmonella enterica subsp. enterica serovar Schwarzengrund str. CVM19633 (CP001127)
Salmonella enterica serovar Typhi strain CT18 (AL627279)
Salmonella enterica subsp. enterica serovar Typhi str. P-stx-12 (CP003278)
Salmonella enterica subsp. enterica serovar Typhi Ty2 (AE014613)
Citrobacter rodentium ICC168 (FN543502)
Klebsiella oxytoca KCTC 1686 complete genome (CP003218)
Klebsiella pneumoniae subsp. pneumoniae HS11286 (CP003200)
Enterobacter cloacae SCF1 (CP002272)
Staphylococcus aureus (NC009632)

FIGURE S2

(A)

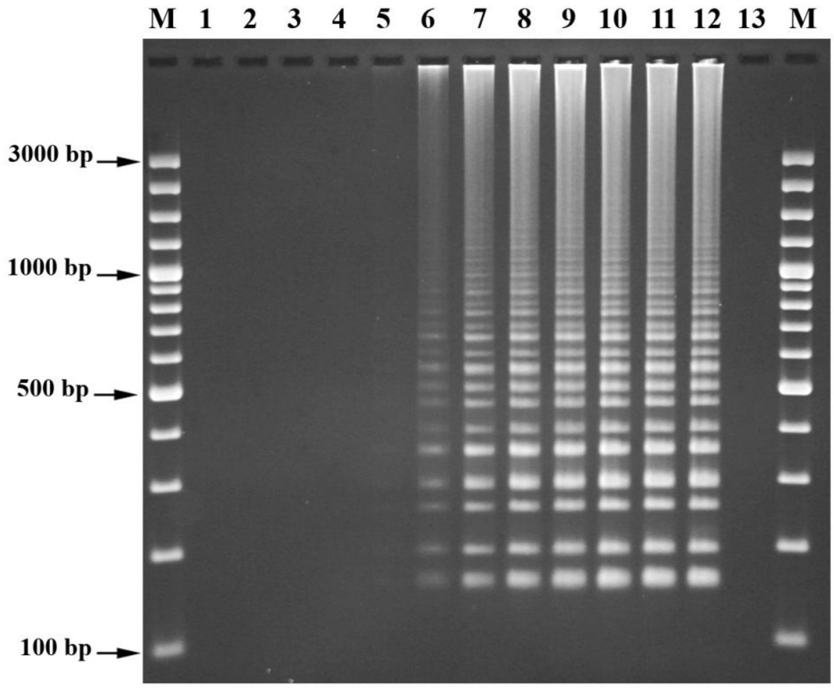
M 1 2 3 4 5 6 7 8 9 10 11 12 13 M
3000 bp
1000 bp
500 bp
100 bp

(B)

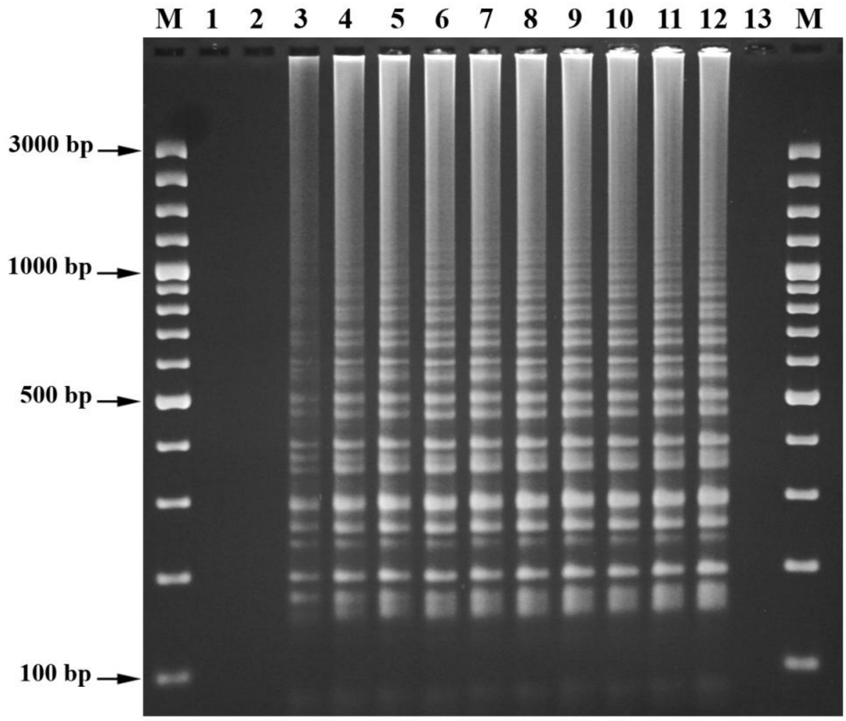
M 1 2 3 4 5 6 7 8 9 10 11 12 13 M
3000 bp
1000 bp
500 bp
100 bp

FIGURE S3

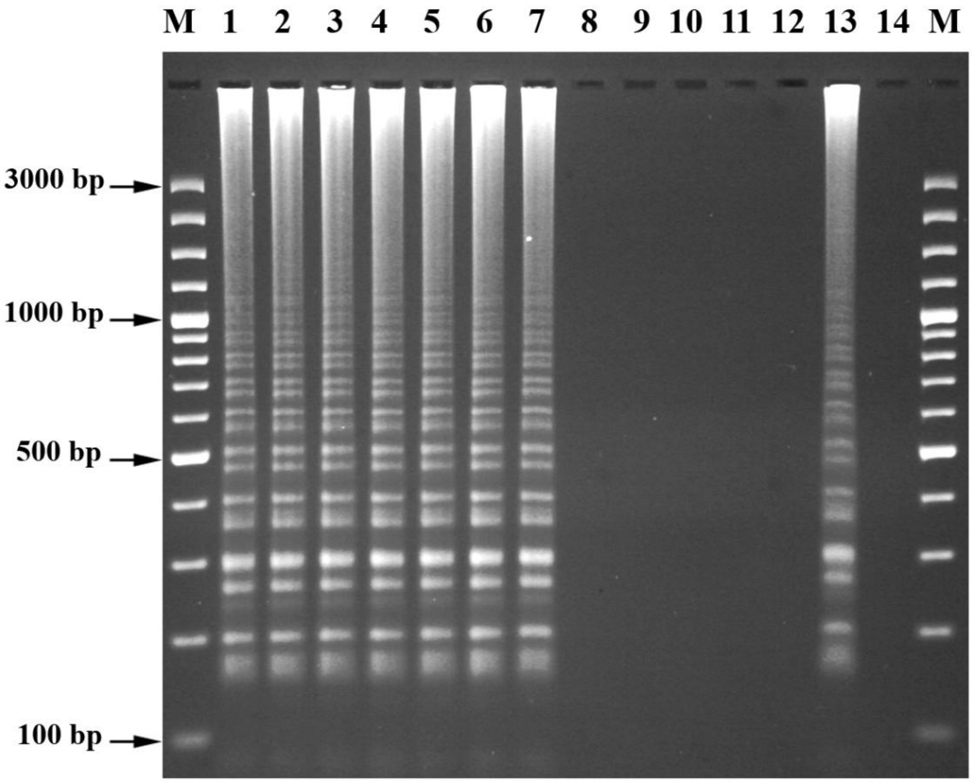

FIGURE S4

(A)

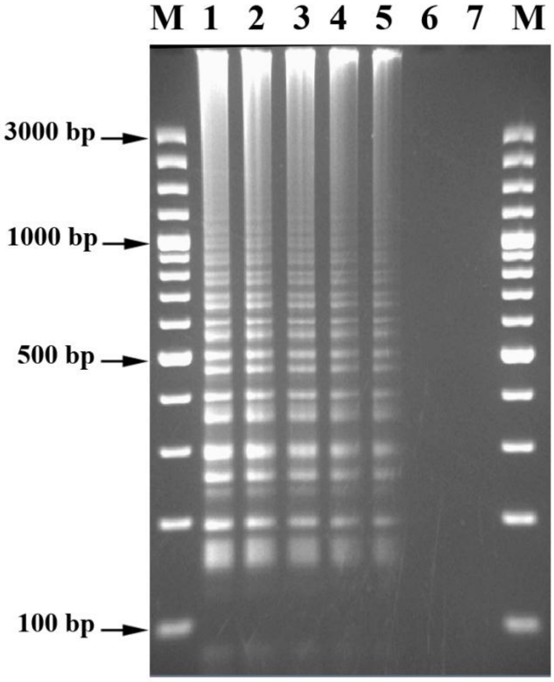
M 1 2 3 4 5 6 7 M
3000 bp
1000 bp
500 bp
100 bp

(B)

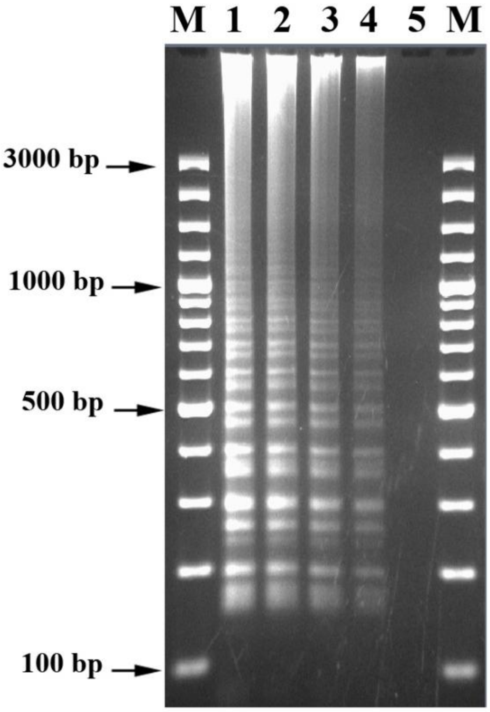
M 1 2 3 4 5 M
3000 bp
1000 bp
500 bp
100 bp

FIGURE S5
